# Honokiol Inhibits HIF-1α-Mediated Glycolysis to Halt Breast Cancer Growth

**DOI:** 10.3389/fphar.2022.796763

**Published:** 2022-03-08

**Authors:** Xianglan Yi, Mengxin Qi, Mingxiang Huang, Sheng Zhou, Jing Xiong

**Affiliations:** Institute of Pathology, Tongji Hospital, Tongji Medical College, Huazhong University of Science and Technology, Wuhan, China

**Keywords:** breast neoplasms, glycolysis, HIF-1α, HNK, ubiquitination

## Abstract

**Background:** Hypoxia-inducible factor-1α (HIF-1α) induces the expression of glycolysis-related genes, which plays a direct and key role in Warburg effect. In a recent study, honokiol (HNK) was identified as one of the potential agents that inhibited the HIF-1α signaling pathway. Because the HIF- 1α pathway is closely associated with glycolysis, we investigated whether HNK inhibited HIF-1α-mediated glycolysis.

**Methods:** The effects of HNK on HIF-1α-mediated glycolysis and other glycolysis-related genes’ expressions, cancer cells apoptosis and tumor growth were studied in various human breast cancer models *in vitro* and *in vivo*. We performed the following tests: extracellular acidification and oxygen consumption rate assays, glucose uptake, lactate, and ATP assays for testing glycolysis; WST-1 assay for investigating cell viability; colony formation assay for determining clonogenicity; flow cytometry for assessing cell apoptosis; qPCR and Western blot for determining the expression of HIF-1α, GLUT1, HK2 and PDK1. The mechanisms of which HNK functions as a direct inhibitor of HIF-1α were verified through the ubiquitination assay, the Co-IP assay, and the cycloheximide (CHX) pulse-chase assay.

**Results:** HNK increased the oxygen consumption rate while decreased the extracellular acidification rate in breast cancer cells; it further reduced glucose uptake, lactic acid production and ATP production in cancer cells. The inhibitory effect of HNK on glycolysis is HIF-1α-dependent. HNK also downregulated the expression of HIF-1α and its downstream regulators, including GLUT1, HK2 and PDK1. A mechanistic study demonstrated that HNK enhanced the self-ubiquitination of HIF-1α by recruiting two E3 ubiquitin ligases (UFL1 and BRE1B). *In vitro,* HNK inhibited cell proliferation and clonogenicity, as well as induced apoptosis of cancer cells. These effects were also HIF1α-dependent. *In vivo*, HNK inhibited tumor growth and HIF-1α-mediated glycolysis.

**Conclusion:** HNK has an inhibitory effect on HIF-1α-mediated glycolysis in human breast cancer. Our research revealed a new mechanism of HNK as an anti-cancer drug, thus representing a novel strategy to improve the prognosis of cancer.

## Introduction

Most eukaryotic cells can cope with environmental changes by changing their metabolic strategies (Seyfried et al., 2014). For example, cancer cells can inhibit the signaling of the mitochondrial aerobic respiratory chain and increase glycolysis to maintain ATP production levels (Kroemer and Pouyssegur, 2008). Even in a normal oxygen environment, tumor cells mainly rely on glycolysis for glucose metabolism -- Warburg effect (Warburg, 1956; Vaupel et al., 2019). Glycolysis allows cancer cells to have a faster production rate, meet their enhanced metabolic needs, and reduce the effect of hypoxia on ATP synthesis (Dias et al., 2019; Abbaszadeh et al., 2020). It can also inhibit mitochondrial function, reduce the production of reactive oxygen species, and promote the survival of cancer cells by increasing the threshold of apoptosis. *Cancer* cells can switch from their aerobic metabolism to the glycolytic pathway by increasing the expression of hypoxia-inducible factor-1α (HIF-1α) (Courtnay et al., 2015). Specifically, HIF-1α accumulated in tumor cells is dimerized with HIF-1β, which binds to the hypoxia response element (HRE) in the transcription regulation region of the glucose transporter or other metabolic rate-limiting enzyme genes to promote their expression, resulting in reprogramming of cancer cell metabolism (Semenza, 2011a; Semenza, 2011b). First, the accumulation of HIF-1α can induce the expression of glucose transporter-1 (GLUT1) and enhance glucose uptake by cancer cells (Lunt and Vander Heiden, 2011). Second, HIF-1α promotes glucose phosphorylation and increases the synthesis of glucose-6p (G6P) by upregulating the level of hexokinase-2 (HK2) (Ghashghaeinia et al., 2019). It can also induce pyruvate dehydrogenase kinase-1 (PDK1) to phosphorylate pyruvate dehydrogenase (PDH) (Masoud and Li, 2015), resulting in pyruvate accumulation and lactic acid increase (Kim et al., 2006; Semenza, 2013). In addition, HIF-1α can provide sufficient nucleotides for hypoxic cancer cells by promoting anabolism, including the pentose phosphate pathway, to meet their rapid growth needs (Hayes et al., 2020). Therefore, in view of the core role of HIF-1α in the metabolic reprogramming of cancer cells (Song et al., 2020), which makes it an ideal target for cancer metabolic treatment, this study focuses on HIF-1α and attempts to intervene glycolysis of cancer cells by directly inhibiting its expression.

Natural compounds are combinations of compounds with unique chemical structures. Compared with standard chemotherapy drugs, natural compounds are less toxic and have fewer side effects, making them a promising source of ideal anticancer drugs. This paper focuses on the natural product honokiol (HNK) (Yi et al., 2021) (Supplementary Figure S1), a natural phenolic compound from Magnolia, which is often used for anti-inflammatory and antioxidant treatment (Banik et al., 2019). Some studies have reported the inhibitory effects of HNK on HIF-1α and hypoxia-related signaling pathways (Vavilala et al., 2012; Vavilala et al., 2014). Therefore, this study used breast cancer as the research object to explore whether HNK can also affect the glucose metabolism of breast cancer cells through HIF-1α and its possible regulatory mechanism. Studies have found that HNK is a promising anti-tumor compound. By regulating NF-κB, STAT3, EGFR, mTOR, VEGF and other signaling pathways, it exerts a wide range of anti-cancer effects, including cancer cell cycle arrest, retardation and apoptosis, migration, epithelial-mesenchymal transition, inhibiting cancer cells proliferation, invasion and tumor angiogenesis (Samarajeewa et al., 2013; Rauf et al., 2018). However, the regulatory role of HNK in the metabolism of tumor cells has not yet been reported. This study preliminarily explores the mechanism by which HNK interferes with glycolysis in breast cancer cells by inhibiting HIF-1α expression. We found that HNK significantly downregulated HIF-1α protein levels in breast cancer cells but had no evident inhibitory effect on its mRNA. Therefore, we speculate that HNK regulation of HIF-1α occurs during protein translation or post-translational protein modification.

Degradation by ubiquitin proteasome is the main mechanism of post-translational regulation of the HIF-1α protein. Under normoxic conditions, HIF-1α ubiquitination occurs through the proline 4-hydroxylase (PHD)/von Hippel- Lindau E3 ubiquitin ligase (pVHL) pathway (Liu et al., 2020). After HIF-1α is hydroxylated by PHD, pVHL recognizes and binds, and is subsequently degraded by 26S proteasome (Andersen et al., 2011). However, PHD activity is reduced under hypoxic conditions, and HIF-1α cannot be hydroxylated, leading to its accumulation in cells. According to reports, heat shock proteins can participate in the ubiquitination and degradation of HIF-1α. For example, the binding of HSP70 to the ubiquitin ligase CHIP promotes the degradation of HIF-1α. When HSP70 is lacking, HSP90β and receptor protein kinase C1 (RACK1) compete with HIF-1α and promote its degradation through ubiquitination (Luo et al., 2010). In this study, we found that HNK reduced the accumulation of the HIF-1α protein in breast cancer cells by improving the ubiquitination degradation of HIF-1α. However, unlike the regulatory pathway reported above, we detected through mass spectrometry that HNK recruited two E3 ubiquitin ligases, UFL1 and BRE1B, which significantly improved the ubiquitination level of HIF-1α. The degradation of HIF-1α accelerates the reversal of the expression of glycolysis-related proteins GLUT1, PDK1, and HK2 in cancer cells, which weakens the glycolysis of breast cancer cells and indirectly leads to tumor growth inhibition. This study has discovered a new mechanism of the anticancer effect of HNK, which has recently gained attention in the field of tumor metabolism research, thus paving a new direction for cancer treatment.

## Materials and Methods

### Cell Lines and Reagent

Two human breast cancer cell lines (MCF-7 and MDA-MB-231) was obtained from the China Center for Type Culture Collection. In addition, HNK was purchased from Sigma-Aldrich, and the stock solution of that was diluted in dimethyl sulfoxide (DMSO) at a concentration of 10 mM. At different concentrations (0, 10, 20, 30, and 40 μM) and different times (2, 4, 8, and 24 h), the same volume of DMSO was used as a carrier control to determine the *in vitro* biological activity of HNK.

### Plasmids and Gene Transfection

We ligated the full-length HIF-1α sequence to the pcDNA3.1 vector to construct an overexpression plasmid, which was called pcDNA-HIF-1α. The breast cancer cells were transfected with pcDNA- HIF-1α and empty vector as previously described. Cell transfection was conducted when the confluency of cells (MCF-7, MD-MBA-231) is up to 70–80%, according to the manufacturer’s instructions. We diluted 2 μg plasmid and 4 μl Lipofectamine 3,000 (Thermo) in 0.2 ml Opti- MEM medium (Gibco). Next, the cells were kept at 37°C for 6–8 h, and then the mixture was replaced with 2 ml DMEM complete medium. The overexpression rate was detected by Western blotting and qRT-PCR after 48 h.

According to the manufacturer’s protocol, siRNA (Invitrogen) knockdown the cells were transiently transfected with BRE1B siRNA (GCA​AGA​AGA​TCG​CGG​ATG​A), UFL1 siRNA (GTT​CCA​ACA​TCG​ACA​AGC​A) or control siRNA (scrambled sequence) by using RNAi Max transfection reagent (Invitrogen). Briefly, BRE1B siRNA or UFL1 siRNA in 50 nM was transfected into MCF-7 and MD-MBA-231 with Lipofectamine 3,000 (Thermo) for 24 h before HNK stimulation. The knockout effect was detected by Western blot and qRT-PCR after 24 h.

### Quantitative RT-PCR

Quantitative RT-PCR was performed to measure the mRNA expressions level of HIF-1α, HK2, PDK1, and GLUT1. The primer sequences were as follows: HIF-1α forward: GAA​CGT​CGA​AAA​GAA​AAG​TCT​CG; HIF-1α reverse: CCT​TAT​CAA​GAT​GCG​AAC​TCA​CA; HK2 forward: GAG​CCA​CCA​CTC​ACC​CTA​CT; HK2 reverse: CCA​GGC​ATT​CGG​CAA​TGT​G; PDK1 forward: CTG​TGA​TAC​GGA​TCA​GAA​ACC​G; PDK1 revers: TCC​ACC​AAA​CAA​TAA​AGA​GTG​CT; GLUT1 forward: GGC​CAA​GAG​TGT​GCT​AAA​GAA; GLUT1 reverse: ACA​GCG​TTG​ATG​CCA​GAC​AG; ACTIN forward: CAT​GTA​CGT​TGC​TAT​CCA​GGC; ACTIN reverse: CTC​CTT​AAT​GTC​ACG​CAC​GAT.

### Western Blot

Western blot was performed to measure protein expression levels of HIF-1α, PDK1, GLUT1, HK2, Ubiquitin, UFL1, and BRE1B. And following antibodies were used: anti- HIF-1α antibody (1:300; Proteintech, United States), anti-GLUT1 antibody (1:1,000; Proteintech, United States), anti-HK2 antibody (1:2000; Cell Proteintech, United States), anti-PDK1 antibody (1:2000; Proteintech, United States), anti-ubiquitin antibody (1:1,000; Proteintech, United States), anti-UFL1 antibody (1:1,000; Bethl Laboratories, United States) and anti- BRE1B antibody (1:1,000; Bethl Laboratories, United States).

### Co-IP Assay

According to the manufacturer’s protocols, ice-cold IP lysis/wash buffer was used to lyse cells at 4°C for 6 min, following cell lysates were collected by centrifugation (12,000 rpm, 15 min). In the presence of protein A/G magnetic beads, the samples containing equal amount of protein were incubated with IgG or anti- HIF-1α antibody on a rotator for 12 h. After incubation, the protein A/G magnetic beads were thoroughly washed with IP Lysis/Washing Buffer, and the protein was eluted by boiling in 5 × SDS loading buffer before SDS-PAGE.

### Ubiquitination Assay

The breast cancer cells were stimulated with MG132 (30 μM) for 8 h before harvesting. Next, IP lysis/wash buffer was used to lyse cells that attached on plate and then collecting supernatant. After low-temperature centrifugation, supernatant was incubated with protein A/G magnetic beads and anti- HIF-1α for antibody on a rotator at low temperature overnight. Finally, anti-ubiquitin antibody was used for Western blot analysis of the ubiquitinated product.

### Cycloheximide (CHX) Pulse-Chase Assay

CHX pulse-chase assay combined with a standard protein synthesis inhibitor was performed to test the influence of HNK on the turnover of HIF-1α protein. Breast cancer cells treated with HNK or control vehicle were stimulated with 60 μg/ml cycloheximide for different time points. Finally, as described above, protein was extracted and analyzed with Western blot.

### Cross-Linking Co-IP/MS

After stimulation with HNK for 1 h, the breast cancer cells were lysed with RIPA buffer containing protease inhibitors for 35 min on ice, followed by brief ultrasonication. Next, the cell lysate was centrifuged at 12,000 rpm for 25 min at 4°C, and the supernatant was collected and precleared with 15 μl protein A/G magnetic beads. Next, adding 1 μg anti- HIF-1α antibody to the l lysate and incubate at 4°C for 4 h, then incubate with 20 μl protein A/G magnetic beads for 1 h. The beads were washed 5 times with ice-cold IP Lysis/Wash buffer and boiled in 6 0 μl SDS loading buffer for 15 min.

Then, the protein lysates were separated by SDS-PAGE and observed by Coomassie staining. HIF-1α - containing strips were reacted with 15 mM dithiothreitol (DTT) at 25°C for 1 h, basal with 50 mM iodoacetamidane (IAA) for 45 min under dark conditions, and digested by trypsin enzyme. The polypeptides extracted from the gel were analyzed by Orbitrap Elite LC-MS/MS (Thermo, United States). Finally, Proteome Discoverer 1.4 (Thermo) was used to retrieve the accepted MS/MS data in uniprot-Homo sapiens. fasta for in-depth identification and analysis.

### WST-1 Assay

WST-1 assay was performed to determine the cytotoxicity of HNK. According to the manufacturer’s protocols, breast cancer cells were cultured in 96-well plates in the presence of various concentrations of HNK for 24 h. Then, WST-1 reagent (25 μg/well) was added and incubation continued for an additional 5 h. Next, microplate reader (BioTek Instruments) was used to obtain OD value of the wells. Controls wells lacking cells were used to determine background absorbance.

### Colony Formation Assay

Colony formation assay was used to determine clonogenicity of single breast cancer cell, which were transfected with control or pcDNA-HIF-1α subsequently treated with the HNK (30 μM). Breast cancer cells were cultured in 6-well plates with a concentration of 300 cells/well. About 2 weeks later, colonies were visible to the naked eye. Next, the 6-well plates were washed three times with PBS and single cell colony was fixed with 4% paraformaldehyde for 20 min, and then stained with Giemsa solution for 10 min.

### Flow Cytometry

Flow cytometry was used to detect the ability of HNK to induce cell apoptosis. Before the machine test, stain cells with or without HNK treatment with fluorescein isothiocyanate (FITC) -annexin V and BD Pharmingen (BD Pharmingen). The stained cells were then analyzed by CellQuest software (BD Biosciences) following detected *via* FACScan flow cytometer.

### Extracellular Acidification (ECAR) and Oxygen Consumption Rate (OCR) Assays

ECAR and OCR were measured according to the production protocols of the Seahorse XF glycolysis stress kit and Seahorse XF cell water to stress kit, respectively. The results were detected by Seahorse XFe 96 Extracellular Flux Analyzer (Seahorse Bioscience), and the data obtained were evaluated by Seahorse XF-96 Wave software. OCR is expressed in pmols/minute, and ECAR is expressed in mpH/minute.

### Glucose Uptake, Lactate, and ATP Assays

According to the manufacturer’s protocol, ATP production, glucose uptake and lactate production were determined to use ATP Colorimetric Assay kit (Beyotime Biotechnology, China), Glucose Uptake Colorimetric Assay kit (Promrga) and Lactate Assay kit (Beyotime Biotechnology, China), respectively. For colorimetric determination of glucose uptake, briefly, cells were cultured in 96-well plates with a concentration of about 1 × 10^6^ cells/well. Following attaching on plate, breast cancer cells were starved by treated with 100 μl Krebs Ringer phosphate Hepes buffer containing 2% BSA for 50 min. And then adding 10 ul 2-DG (10 mM) and incubation continued for an additional 20 min.

### Xenograft Model

Specific pathogen-free (SPF) nude mice (BALB/c, 18 g, 4 weeks old) were housed in a laminar flow at 28°C under sterile conditions. MCF-7 cells (1×10^6^ cells in 150 μl PBS) were injected subcutaneously into the armpit of nude mice. Ten days after the breast tumor cells were injected, palpable tumors were observed in nude mouse armpit subcutaneous, meaning that a breast cancer xenograft model was established. Then, HNK (diluted in DMSO, 25 mg/kg/day) or same amount of DMSO was administered intraperitoneally for 4 weeks, including tumors volume were measured each day. After drug treatment, the animals were sacrificed, subcutaneous tumor was isolated and final tumor volume and weight were measured to establish a tumor growth curve and tumor tissues were used for Western blot and other analyses.

### Micro Positron Emission Tomography (microPET)/Computed Tomography (CT)

The SUVmax value of the breast tumors, which indicated the glucose uptake of tumor cells, was detected using microPET/CT and was used to determine the glucose metabolism level in breast tumor cells in the xenograft model.

### Statistical Analysis

For each step, at least four independent experiments were conducted. All data was analyzed with mean ± SEM. Using the 2-^ΔΔ^CT method to analyze relative gene expression data (Livak and Schmittgen, 2001). One-way analysis of variance (ANOVA) or the non-parametric Kruskal–Wallis test were introduced to determine the statistical significance of differences between experiment and control groups. All statistical tests are bidirectional, and the significance level is set to *p* < 0.05.

## Results

### HNK Inhibits Glycolysis and Expression of Glycolysis-Related Molecules in Breast Cancer Cells

**FIGURE 1:**
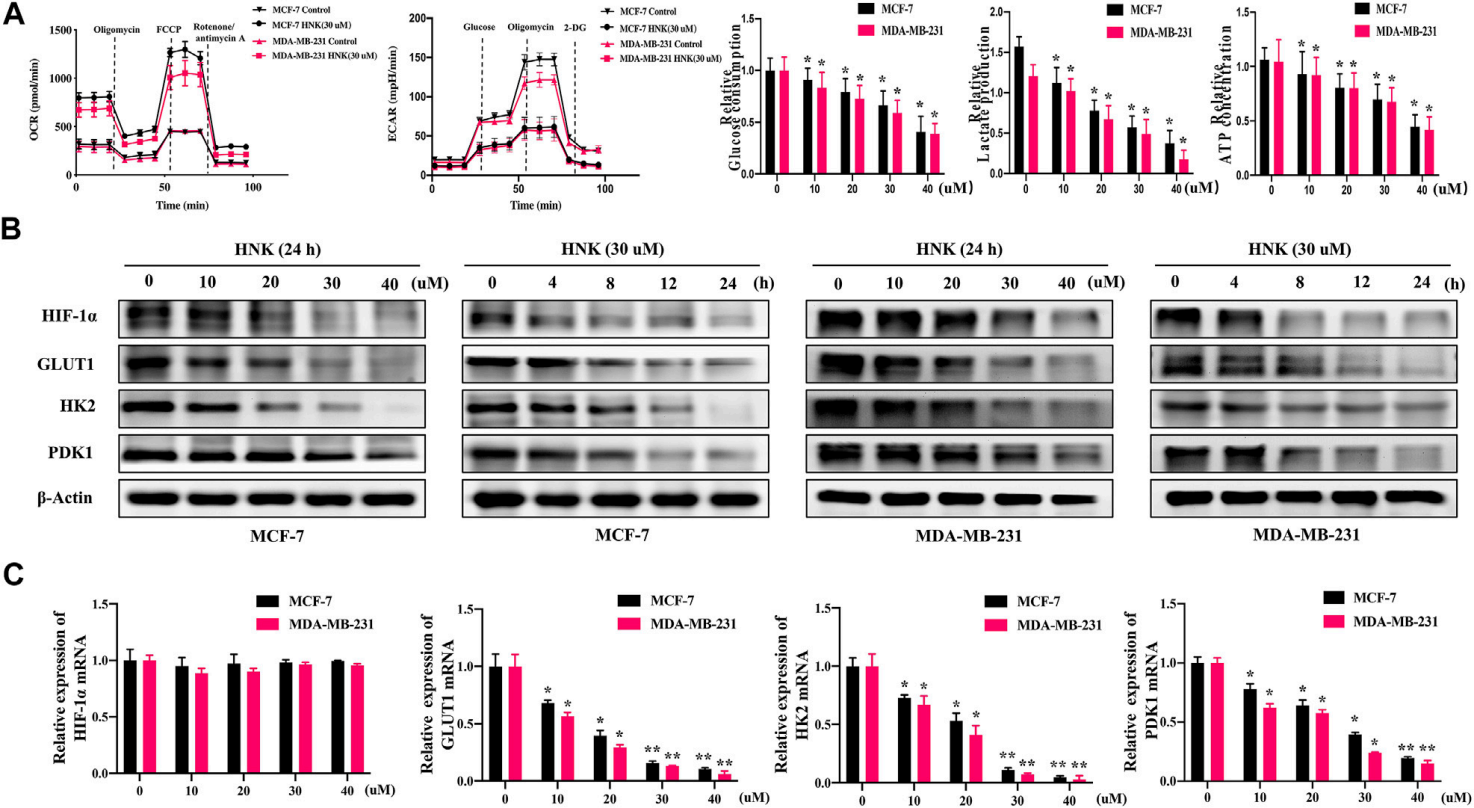
HNK down-regulates glycolysis and related key factors of breast cancer cells. **(A)** HNK (30 μM, 24 h) decreased extracellular acidification rate (ECAR), while increased oxygen consumption rate (OCR) of two breast cancer cell lines (MCF-7 and MDA-MB-231). MCF-7 and MDA-MB-231 cells were treated with increasing concentrations of HNK (0, 10, 20, 30, 40 μM) for 24 h, and then glucose uptake, lactate production, and ATP production were detected. *p < 0.05 compared with 0 μM control group. **(B)** MCF-7 and MDA-MB-231 cells were treated with different concentrations of HNK (0, 10, 20, 30, 40 μM) for 24 h and 30 μM HNK for 0, 4, 8, 12, 24 h respectively. The expressions of HIF-1α, GLUT1, HK2 and PDK1 were detected by Western blot. β-Actin served as an internal control for equal protein loading. **(C)** MCF-7 and MDA-MB-231 cells were treated with different concentrations of HNK (0, 10, 20, 30, 40 μM) for 24 h. Quantitative RT-PCR analysis indicated that HNK down-regulated mRNA expression of GLUT1, HK2 and PDK1 in a dose-dependent manner, while did not affect mRNA expression of HIF-1α. Data represent percentage of the total amount of corresponding mRNA for each fraction. *p < 0.05 and **p < 0.01 compared with 0 μM control group.

First, to explore whether HNK can inhibit glycolysis in breast cancer cells, we detected the changes in OCR, ECAR, glucose consumption, lactic acid production and ATP production in two human breast cancer cell lines (MCF-7 and MDA-MB-231) after HNK intervention. The results showed that after HNK treatment, OCR increased and ECAR decreased. In addition, HNK decreased glucose uptake, lactic acid production and ATP production in cancer cells (Figure 1A).

Second, we observed the effect of HNK on the expression of HIF-1α and its downstream molecules GLUT1, HK2 and PDK1 by qPCR and Western blot experiments, respectively. The results showed that HNK downregulated the protein expression of HIF-1α, GLUT1, HK2 and PDK1 in a dose-and time-dependent manner (Figure 1B). Interestingly, qPCR showed that HIF-1α mRNA levels did not change significantly after HNK treatment, but significantly inhibited the mRNA levels of GLUT1, HK2 and PDK1 (Figure 1C).

### HNK Inhibits the Glycolysis of Breast Cancer Cells Dependent on HIF- 1α

**FIGURE 2:**
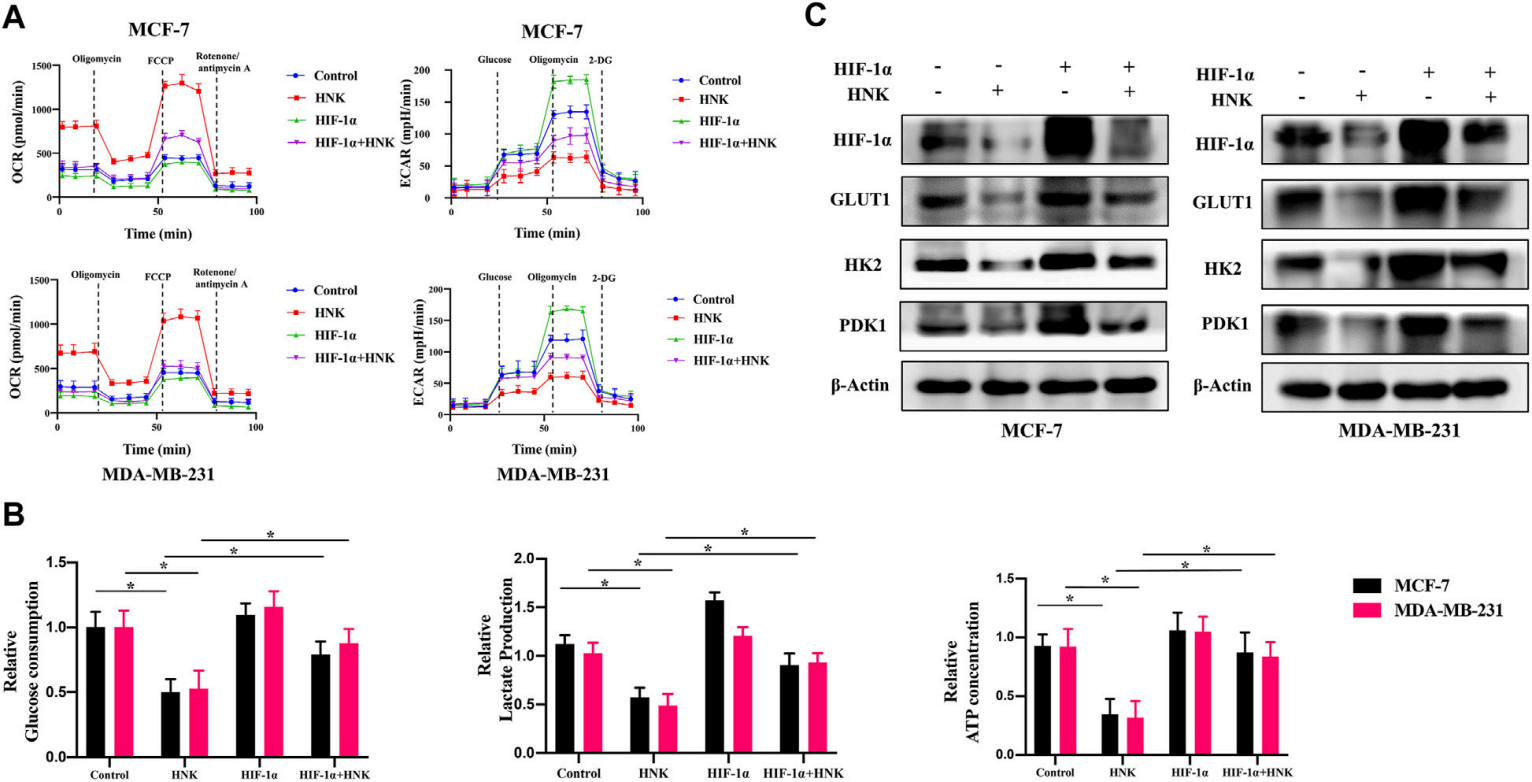
HNK inhibits glycolysis related key factors expression by down-regulating HIF-1α. **(A)** MCF-7 and MDA-MB-231 cells were transfected with HIF-1α overexpression plasmid, and then the tumor cells were incubated with 30 μM HNK for 12 h followed by cell ECAR and OCR tests. Cells with HIF-1α overexpression were less responsive to HNK-induced glycolysis inhibition than control cells. **(B)** MCF-7 and MDA-MB-231 cells were transfected with control or HIF-1α overexpression plasmid, and then the tumor cells were incubated with 30 μM HNK for 12 h followed by glucose uptake, lactate production, and ATP production activities analysis. HNK significantly inhibited glucose uptake, lactate production, and ATP production of MCF-7 and MDA-MB-231 cells, which were rescued by HIF-1α overexpression. *p < 0.05. **(C)** Western blot assays show HIF-1α-overexpression reversed HNK-induced downregulation of GLUT1, HK2 and PDK1. β-Actin served as an internal control for equal protein loading.

To further verify whether HNK inhibits glycolysis in breast cancer cells depending on HIF-1α, we constructed HIF-1α overexpressing breast cancer cells, and compared OCR, ECAR, glucose uptake, lactic acid activities and ATP production between wild-type and HIF-1α overexpressed breast cancer cells after HNK treatment. The results showed that HNK downregulated the level of HIF- 1α protein in both cells, which resulted in an increase in OCR and a decrease in ECAR, glucose uptake, lactic acid production and ATP production. However, compared with that in wild-type breast cancer cells, the role of HNK in HIF-1α overexpressed breast cancer cells was weakened. These results indicate that the inhibitory effect of HNK on glycolysis in breast cancer cells depends on HIF-1α (Figures 2A,B).

In addition, the expression of key glycolysis genes GLUT1, HK2 and PDK1 was detected in breast cancer cells overexpressing HIF-1α after HNK treatment. The results showed that HIF-1α was only downregulated at the protein level, while the protein and mRNA levels of GLUT1, HK2 and PDK1 decreased. However, compared with that in wild-type breast cancer cells, HNK had a weaker inhibitory effect on these genes in breast cancer cells overexpressing HIF-1α (Figure 2C).

### HNK Induces HIF-1α Ubiquitination and Degradation Through UFL1 and BRE1B

**FIGURE 3:**
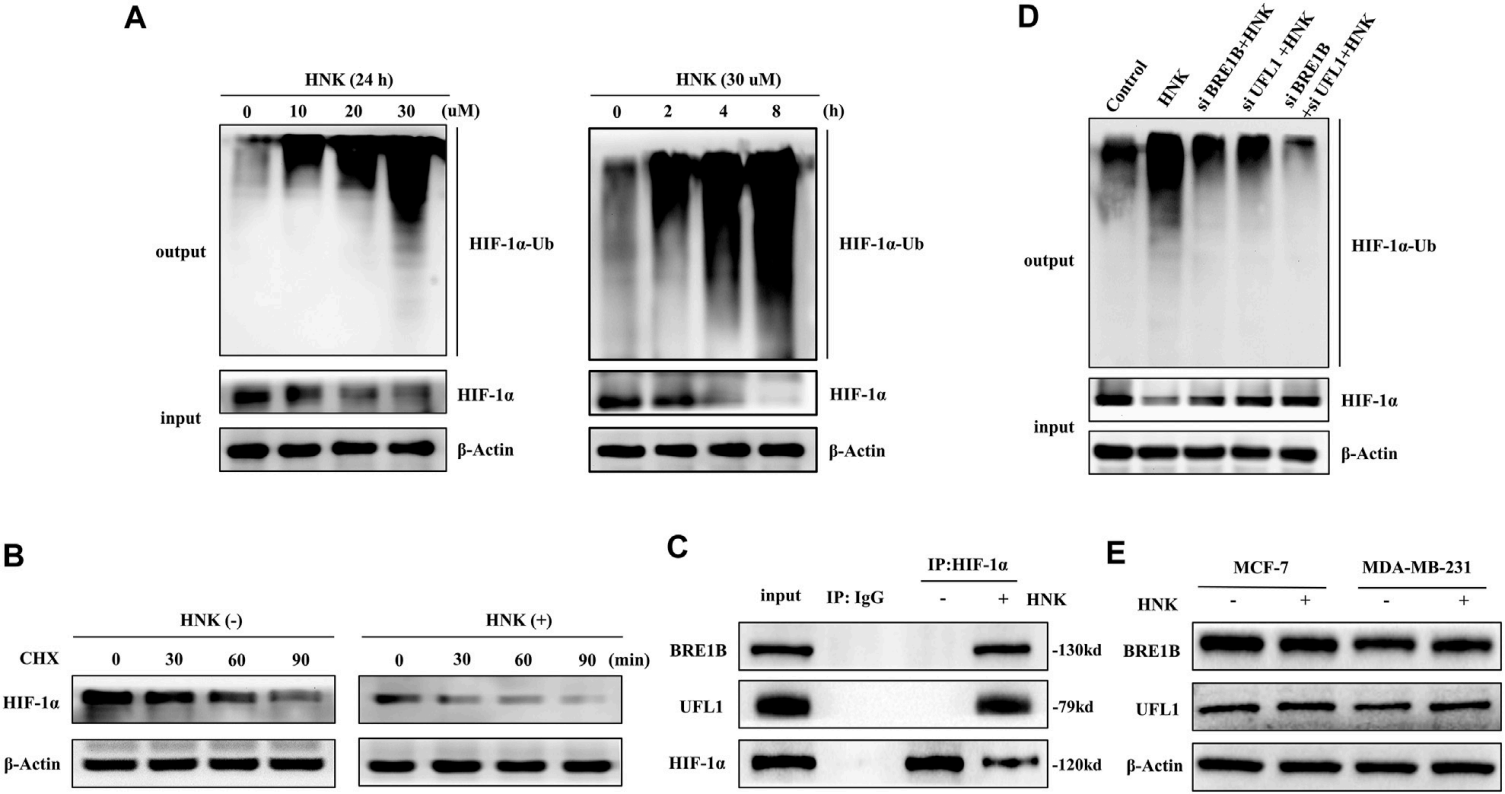
HNK induces HIF-1α ubiquitination and protein-degradation, which requires E3 ubiquitin ligases BRE1B and UFL1. **(A)** MCF-7 cells were treated with different concentrations of HNK for 8 h or 30 μM HNK for indicated times. Ubiquitination assay was performed in MCF-7 cells for testing the dose-response and time-course of HNK on ubiquitination of HIF-1α. **(B)** MCF-7 cells were treated with or without 30 μM HNK for 4 h, followed by addition of a protein synthesis inhibitor cycloheximide (CHX, 50 μg/ml). At different time points after CHX, cell lysates were prepared and analyzed by Western blot assay. **(C)** MCF-7 cells were treated with 30 μM HNK for 3 h. Total cell lysate (500 μg) was immunoprecipitated with anti-HIF-1α antibody or IgG antibody and then subjected to Western blot for indicated proteins. **(D)** MCF-7 cells were transfected with control siRNA, BRE1B siRNA, UFL1 siRNA, and BRE1B siRNA/UFL1 siRNA, followed by 30 μM HNK for 12 h **(E)** MCF-7 and MDA-MB-231 cells were treated with or without 30 μM HNK for 24 h, cell lysates were prepared and analyzed by Western blot assay. β-Actin served as an internal control for equal protein loading.

Since HNK cannot cause the downregulation of HIF-1α expression at the mRNA level, but only significantly reduces its protein expression, we speculate that the regulation of HIF-1α by HNK may be achieved by influencing the post-translational modification of HIF-1α. According to the fact that HIF-1α is degraded mainly by intracellular oxygen-dependent ubiquitin protease degradation under normal oxygen conditions, we speculate that HNK may be involved in the ubiquitination degradation process of HIF-1α, which has been verified by relevant experiments. The results showed that under the action of HNK, the ubiquitination degradation of HIF-1α was enhanced in a dose-and time-dependent manner (Figure 3A). After the CHX experiment, it was found that HNK inhibited HIF-1α through ubiquitination degradation, and the half-life of HIF-1α protein in control cells was approximately 90 min, while the half-life of HIF-1α protein was shortened to less than 30 min after HNK treatment (Figure 3B).

To further understand the specific mechanism of HIF-1α ubiquitination induced by HNK, we performed a mass spectrometric detection of breast cancer with and without HNK intervention (Supplementary Table S1). The results showed that two new E3 ubiquitination ligases UFL1 and BR1B were recruited after HNK stimulation, and the results of Co-IP experiment also showed that UFL1 and BR1B could not combine with HIF-1α before HNK treatment, but obviously combined after adding HNK (Figure 3C). Then, by knocking down UFL1 and BRE1B in turn, the ubiquitination degree of HIF-1α decreased, especially after knocking down both UFL1 and BRE1B, the ubiquitination level of HIF-1α was significantly inhibited (Figure 3D). Finally, we detected the expression levels of UFL1 and BRE1B in various cell lines before and after HNK treatment by Western blotting and found that HNK did not significantly change the expression levels of UFL1 and BRE1B (Figure 3E). Therefore, it is speculated that HNK induces ubiquitination degradation of HIF- 1α by promoting the binding of UFL1 and BRE1B to HIF-1α.

### HNK Inhibits the Growth of Breast Cancer Cells Dependent on HIF- 1α

**FIGURE 4:**
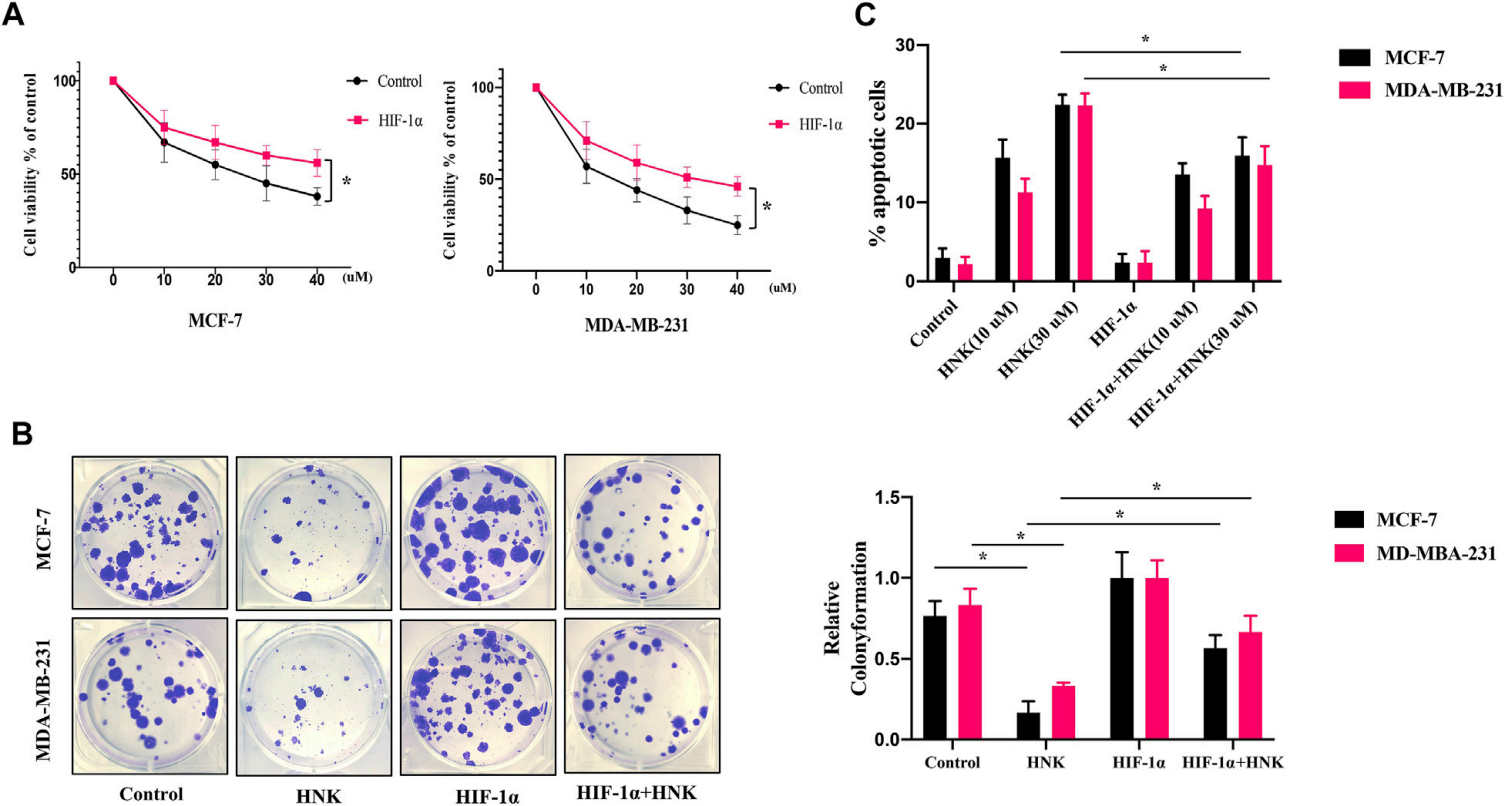
HNK inhibits breast cancer cells growth by down-regulating HIF-1α. MCF-7 and MDA-MB-231 cells were transfected with control or HIF-1α overexpression plasmid, and then the tumor cells were incubated with different concentrations of HNK (0, 10, 20, 30, 40 μM) for 24 h **(A)** WST-1 assay was conducted to compare the cytotoxic effect of HNK between HIF-1α overexpression cells and control cells. HIF-1α overexpression cells were less responsive to HNK-induced cytotoxic effect than control cells. *p < 0.05. **(B)** Colony formation assay was used to determine the inhibitory effect of HNK on the clonogenicity of MCF-7 and MDA-MB-231 cells. Cells were transfected with control or HIF-1α overexpression plasmid subsequently treated with the 30 μM HNK. Number of colonies was calculated after 2 weeks. HNK treatment significantly reduced the colony number of breast cancer cells, which was rescued by HIF-1α overexpression. *p < 0.05. **(C)** MCF-7 and MDA-MB-231 were transfected with control or HIF-1α overexpression plasmid, subsequently treated with HNK (0, 10, 30 μM), and finally analyzed by flow cytometry for apoptosis. HNK significantly induced the apoptosis of breast cancer cells in a dose-dependent manner, and HIF-1α overexpression partially rescued HNK-induced apoptosis. *p < 0.05.

Our research verified that HNK can significantly inhibit glycolysis in breast cancer cells. To further explore whether HNK can inhibit cell viability and whether this effect depends on HIF-1α-mediated glycolysis, MCF-7 and MDA-MB-231 cells were transfected with control or HIF-1α overexpression plasmid, and then the tumor cells were incubated with indicated concentrations of HNK. Compared with control group, the WST-1 test showed that HNK had a significant cytotoxic activity against these breast cancer cells; However, in HIF-1α overexpression cells, the inhibitory effect of HNK on cell viability was weaker than that of control breast cancer cells (Figure 4A). Colony formation experiments also showed that HNK treatment significantly reduced the colony number of breast cancer cells. In addition, results showed that HNK-mediated HIF-1α degradation was required for inhibitory effect of HNK on colony formation of breast cancer cells. HIF-1α overexpression rescued HNK treatment induced reduction of clonal numbers (Figure 4B).

Subsequently, to further explore whether HNK could induce apoptosis of breast cancer cells, HNK treated MCF-7 and MDA-MB-231 cells were stained with annexin V and quantified by flow cytometry. We found that HNK significantly promoted the apoptosis of breast cancer cells in a dose-dependent manner (Figure 4C). In addition, we further explored whether HIF-1α was critical to HNK-induced cell apoptosis and death. Our results indicated HIF-1α indeed participated in HNK induced cell apoptosis and cells with HIF-1α overexpression partially rescued HNK-induced apoptosis of MCF-7 and MDA-MB-231 cells.

### HNK Inhibits Tumor Growth and HIF-1α-Mediated Glycolysis in Breast Cancer Xenograft Model

**FIGURE 5:**
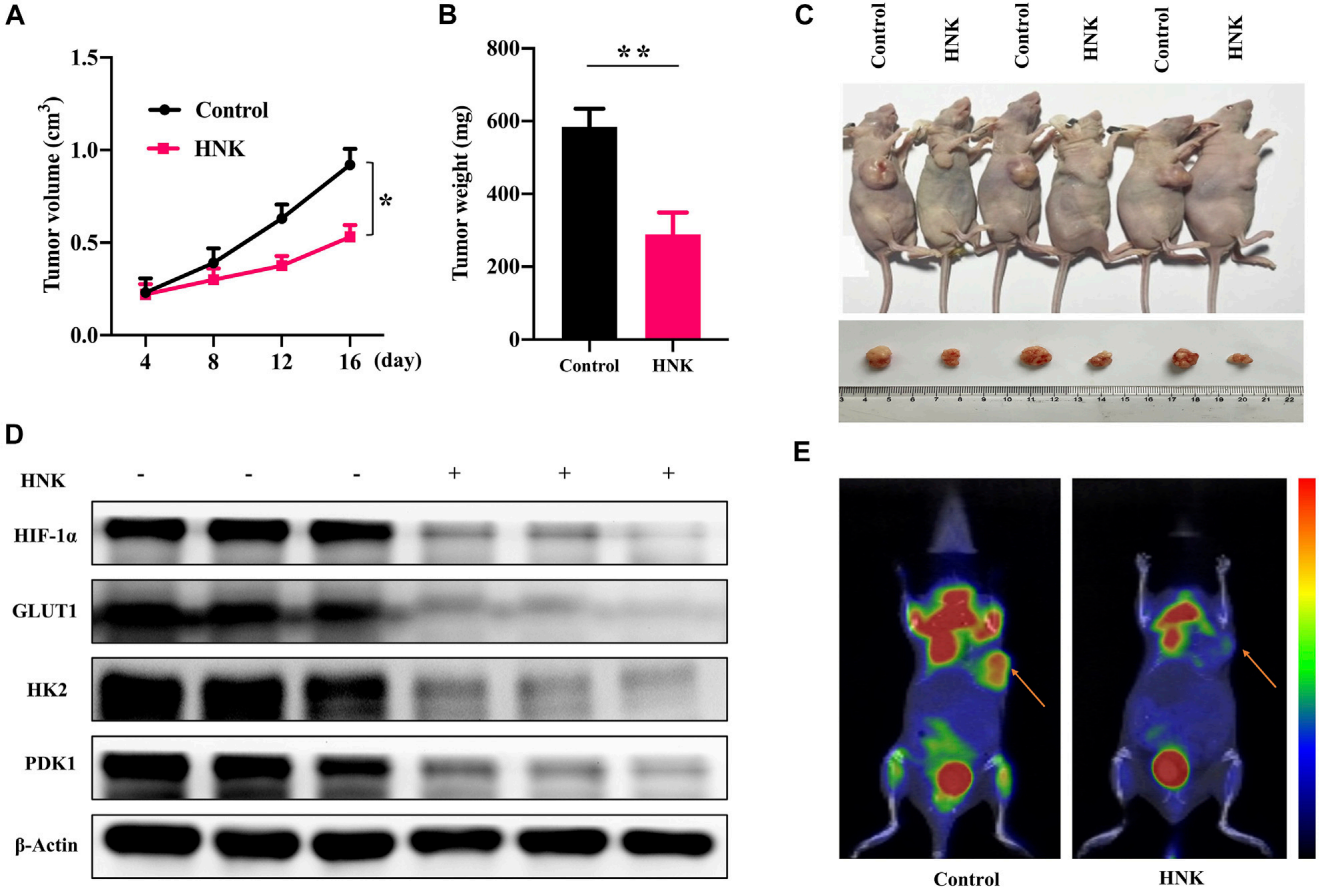
HNK suppresses tumor growth in breast cancer xenograft models. MCF-7 cells were subcutaneously implanted into nude mice. The nude mice were subsequently treated with HNK. All mice underwent monitoring of tumor growth. On day 22 post inoculation, xenograft tumor tissues were obtained for testing the expression of HIF-1α, GLUT1, HK2 and PDK1. **(A)** Xenograft tumor size. *p < 0.05. **(B)** Xenograft tumor weight on day 22. **p < 0.01. **(C)** Representative pictures from each group. **(D)** Expression of HIF-1α, GLUT1, HK2 and PDK1 in tumor tissues determined by Western blot. β-Actin served as an internal control for equal protein loading. **(E)** The micro-PET/CT imaging of glucose metabolism in the vivo tumor tissues on day 16, indicating HNK suppressed glucose metabolism of vivo tumor tissues. Data represent mean ± SEM. *n* = 6 mice/group.

Because HNK can inhibit glycolysis mediated by HIF-1α and the growth of cancer cells, we constructed a nude mouse model of breast cancer transplantation to observe whether HNK can inhibit glycolysis mediated by HIF-1α and slow down tumor growth *in vivo*. After the breast cancer xenograft model was established, HNK was injected intraperitoneally to continuously monitor tumor growth in nude mice within the designed period, and the tumor size and mass were measured after monitoring. The results showed that the tumor size and mass in the HNK treatment group were significantly smaller than those in the control group (Figures 5A–C). The removed tumor tissues were subjected to Western blot, and it was confirmed that the protein expression of HIF-1α and its downstream glycolytic genes GLUT1, HK2 and PDK1 in the HNK treatment group was significantly lower than that in the control group (Figure 5D). MicroPET/CT showed that glucose uptake in the HNK treatment group was significantly inhibited compared with that in the control group (Figure 5E). Therefore, the above-mentioned *in vivo* experiments also show that HNK can inhibit glycolysis by inhibiting the expression of HIF-1α and its downstream GLUT1, HK2, and PDK1, and then slow down tumor growth.

## Discussion

HNK has been widely studied for its classic antitumor effects, such as activation of pro-apoptotic factors (Pan et al., 2016), inhibition of anti-apoptotic proteins and transcription factors, downregulation of various enzymes, chemokines, cell surface adhesion molecules, and cyclins, but its role in metabolic reprogramming is not well understood (Ganapathy-Kanniappan and Geschwind, 2013). Because HNK has an antioxidant effect and HIF-1α is the key molecule of oxygen sensing in cells, we are interested in understanding how HNK and HIF-1α synergistically regulate energy metabolism in cancer cells. Under the condition of accelerating the growth of tumor cells, the metabolic pathway changes from oxidative phosphorylation to the glycolytic pathway, which is mainly realized by upregulating the glycolysis-related proteins GLUT1, HK2, and PDK1 mediated by HIF-1α (Eales et al., 2016). Meanwhile, the upregulation of LDHA mediated by HIF-1α promoted the formation of lactic acid from pyruvate (Hong et al., 2020), which contributed to the acidification of the tumor microenvironment. Our experimental observation results showed that these changes were reversed by the addition of HNK. HNK reduced the expression of HIF-1α, GLUT1, HK2 and PDK1, decreased ECAR, increased OCR, and decreased glucose uptake, lactate production and ATP production in cancer cells. These effects showed that HNK can significantly promote the inhibition of glycolysis in tumor cells. We speculate that this is because HNK reduces the expression of HIF-1α protein, which in turn inhibits the transcription of key molecules GLUT1, HK2, and PDK1 on the glycolysis link, resulting in a decrease in the glycolysis level of cancer cells. Moreover, these interventions of HNK on glycolysis ultimately indirectly inhibited the proliferation of breast cancer cells and slowed the growth of tumors (Hou et al., 2020).

There are some reports on the regulation of HIF-1α by HNK, such as studies of Keng-Lilan et al. (Vavilala et al., 2012) and Divya et al. (Vavilala et al., 2014), which reported that HNK can inhibit the binding of HIF-1α to HRE, which leads to the downregulation of HRE-mediated transcription activity, but HNK has no direct regulation on the expression of HIF- 1α, nuclear translocation, and binding to HIF-1β. In another study, Divya et al. observed that HNK inhibited HIF-1α mRNA levels, but the mechanism was unclear (Lan et al., 2011). In contrast to the conclusions reported above, our current research has found a new phenomenon in which HNK regulates HIF-1α in breast cancer cells, that is, HNK has no obvious effect on the mRNA level of HIF-1α in breast cancer cells, but can significantly reduce the protein level of HIF-1α in cancer cells; that is, it can regulate the stability of translated HIF-1α protein.

**FIGURE 6:**
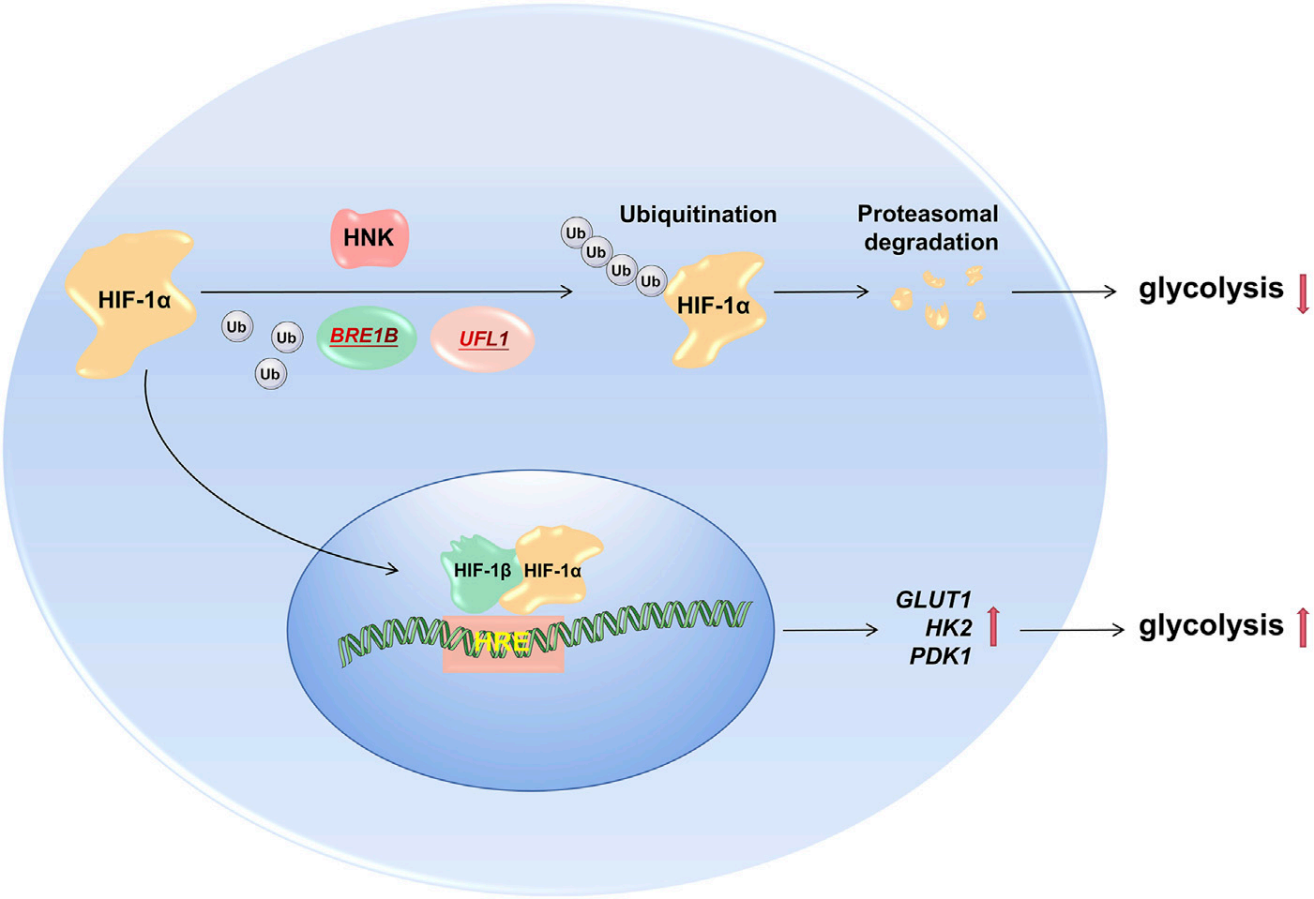
HNK promotes HIF1a degradation by recruiting E3 ubiquitin ligase to inhibit glucose metabolism. Under normal circumstances, tumor cells promote partial HIF-1α degradation through ubiquitin ligase, and the remaining HIF-1α enters the nucleus to promote the transcription of downstream target genes (GLUT1, PDK1, and HK2), and its role includes maintaining tumor cells aerobic glycolysis process; After the drug HNK acts on the cells, it recruits two new E3 ubiquitin ligases (BRE1B, UFL1) to aggravate the degradation of HIF-1α ubiquitination, and its downstream target gene expression also decreased, the glycolysis process of tumor cells is inhibited, so as to achieve the effect of hindering the growth of tumor cells.

Subsequently, we explored the mechanism by which HNK downregulates the expression of HIF-1α. It is well-known that ubiquitination degradation mediated by the E3 ligase pVHL is the main degradation pathway of HIF-1α under normoxic conditions (Fernandez Esmerats et al., 2019). Therefore, HNK also promotes ubiquitination degradation of HIF-1α. First, we explored whether HNK could participate in ubiquitination degradation of HIF-1α. Through the CHX experiment, we proved that the ubiquitination degradation level of HIF-1α was significantly improved under the action of HNK. Then, through mass spectrometry, it was found that HNK recruited two E3 ubiquitin ligases, UFL1 and BRE1B, which directly participated in the ubiquitination degradation process of HIF-1α, which was also verified by a Co-IP experiment. However, the inhibitory effect of HNK on HIF- 1α was weakened after knocking down UFL1 and BRE1B. Therefore, we speculate that UFL1 and BRE1B are also E3 ligases involved in the ubiquitination degradation of HIF-1α, while HNK promotes the ubiquitination degradation of HIF-1α protein by UFL1 and BRE1B in cancer cells. Of course, we only found these two new E3 ligases by mass spectrometry, and whether there are other E3 ligases involved cannot be ruled out. However, this study found that HNK has a definite relationship with UFL1 and BRE1B and can promote the combination of UFL1 and BRE1B with HIF-1α, which improves the ubiquitination degradation level of HIF-1α to a certain extent, and then reverses tumor growth via the glycolytic pathway (Figure 6). This may be another way for HNK to exert its antitumor effects.

In this study, we not only clarified the mechanism by which HNK inhibits the glycolysis of cancer cells but also identified E3 ubiquitin ligase in the ubiquitination process of HIF-1α; however, the mechanism through which HNK promotes the ubiquitination degradation of HIF-1α via UFL1 and BRE1B remains to be further studied—this will help better understand the role of HNK in the energy metabolism of tumor cells.

## Data Availability

The original contributions presented in the study are included in the article/Supplementary Material, further inquiries can be directed to the corresponding authors.
