# Supplementary material for: Honokiol Inhibits HIF-1α-Mediated Glycolysis to Halt Breast Cancer Growth
Source: Front Pharmacol. 2022 Mar 8;13:796763. doi: 10.3389/fphar.2022.796763 (PMC8957822; doi:10.3389/fphar.2022.796763)
Supplement: Supplementary file 1 [file DataSheet1.PDF]

Table S1:All E3 ubiquitin ligases before and after the treatment of HNK were selected based on ms results.

Group	HNK(-)			HNK(+)			
**Accession**	Q14258	O43255	A0A087WWG9	Q14258	O43255	O75150-3	O94874-3
**Description**	E3 ubiquitin/ISG15 ligase TRIM25 OS=Homo sapiens OX=9606 GN=TRIM25 PE=1 SV=2 - [TRI25_HUMAN]	Siah E3 ubiquitin protein ligase 2 OX=9606 GN=SIAH2 PE=1 SV=2 - [O43255_HUMAN]	E3 ubiquitin-protein ligase UHRF1 (Fragment) OS=Homo sapiens OX=9606 GN=UHRF1 PE=1 SV=1 - [A0A087WWG9_HUMAN]	E3 ubiquitin/ISG15 ligase TRIM25 OS=Homo sapiens OX=9606 GN=TRIM25 PE=1 SV=2 - [TRI25_HUMAN]	Siah E3 ubiquitin protein ligase 2 OX=9606 GN=SIAH2 PE=1 SV=2 - [O43255_HUMAN]	Isoform 3 of E3 ubiquitin-protein ligase BRE1B OS=Homo sapiens OX=9606 GN=RNF40 - [BRE1B_HUMAN]	Isoform 3 of E3 UFM1-protein ligase 1 OS=Homo sapiens OX=9606 GN=UFL1 - [UFL1_HUMAN]
**Score**	2.04	1.83	1.72	1.75	0.00	0.00	0.00
**Coverage**	4.76	2.20	2.02	3.81	8.20	2.86	2.16
**Proteins**	1	1	6	1	3	4	3
**Unique Peptides**	2	3	1	2	3	1	1
**Peptides**	2	2	1	2	1	1	1
**PSMs**	2	2	1	2	1	1	1
**AAs**	630	749	496	630	749	838	509
**MW [kDa]**	70.9	74.5	55.0	70.9	74.5	94.7	57.5
**calc. pI**	8.09	5.77	9.51	8.09	5.77	5.58	6.30
