# Supplementary figures and images for: Honokiol Inhibits HIF-1α-Mediated Glycolysis to Halt Breast Cancer Growth
Source: Front Pharmacol. 2022 Mar 8;13:796763. doi: 10.3389/fphar.2022.796763 (PMC8957822; doi:10.3389/fphar.2022.796763)

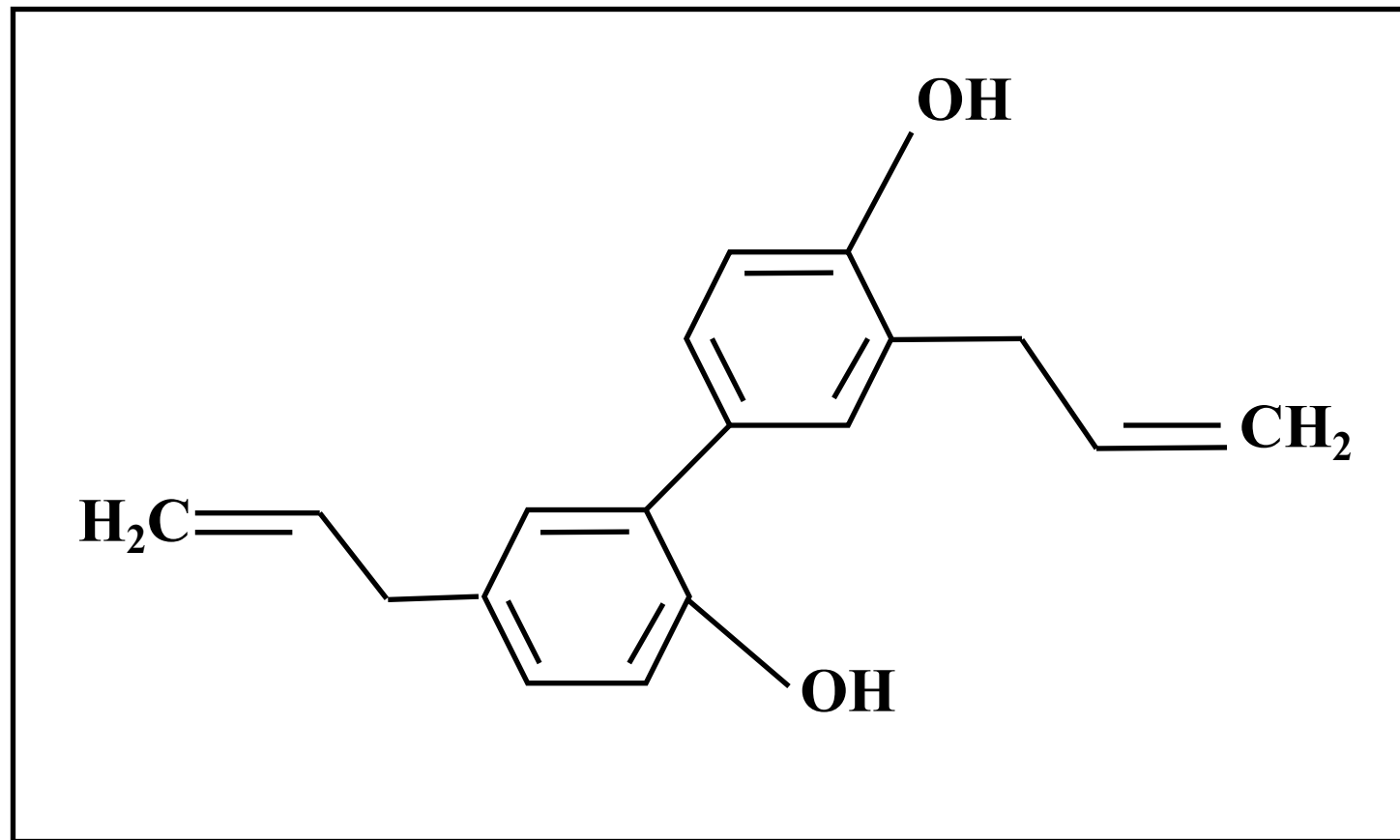

Supplement: Supplementary file 2 [file Image1.PDF]
